# Primary Muscular Tuberculosis
1Professor F. Lejars, M.D., Medical Press and Circular, June 22, 1904.


**Published:** 1904-07-23

**Authors:** 


### PRIMARY MUSCULAR TUBERCULOSIS.[1]

Primary tuberculosis of muscles is a clinical term rather than an exact pathological definition. The tuberculous foci, it is true, are embedded in the muscle and have the general clinical characters of muscle tumours, but their point of origin is the connective tissue sheath, not the muscle. Again, though described as primary to distinguish it from a secondary tuberculous myositis due to extension by contiguity of tissue, the condition probably always follows an initial lesion, which, however, may be so remote and obscure that the tuberculosis of the muscle constitutes for all practical purposes a distinct morbid entity. Three varieties of the disease are described, namely, the gumma, the cold abscess, and the tuberculous infiltration, the last-named being the most infrequent of the three. The cold abscess is the most common form, and it has to be distinguished from hydatid cyst, sarcoma, lipoma, and syphilitic gumma; also it must be remembered that a chronic, indolent, intra-muscular tumour may be a non-tuberculous abscess due to the suppuration of an old hæmatoma, or an abscess containing a culture of the Eberth bacillus and following enteric fever. In other cases there are numerous tumours widely spread over the body and often appearing at or about the same date. The diagnosis in these instances is helped by the different consistence of the several tumours, some being soft and fluctuant, while others are firm and resistant. If necessary, an exploratory puncture will help to distinguish the condition from sarcomatosis, multiple hydatid cysts, and from actinomycosis. In spite of the evidence of widespread infection in these cases, the patients often show little depreciation of the general health, unless at the same time they are the victims of pulmonary or other serious form of tuberculosis. The tumours ought always to be extirpated, whether multiple or single, and the operation should be a thorough one, not mere scraping or curettage, but complete ablation, removing, where necessary, considerable masses of muscle. If left without treatment, these tuberculous nodules tend to perforate the aponeuroses and spread into the subcutaneous tissues, or to run along the tendons and invade the synovial sacs. The operation not only avoids these risks, but is usually followed by improvement of the original focus, should this be obvious and active. The muscles most frequently affected are the so called "working" muscles—that is, those upon which falls a large amount of work, such as the rectus femoris, brachial biceps, supinator longus, deltoid, etc. The tuberculous nodule, it may be noted, not infrequently originates in or near the point where the muscle blends with its tendon. These two facts may be held to suggest that rupture or detachment of the muscular fibres, due perhaps to some slight injury, is an event which determines the point of infection.

[1] Professor F. Lejars, M.D., Medical Press and Circular, June 22, 1904.

